# Wait and Capture: Unwinding the Strategy of a DEAD-Box Helicase

**DOI:** 10.1371/journal.pbio.1001982

**Published:** 2014-10-28

**Authors:** Richard Robinson

**Affiliations:** Freelance Science Writer, Sherborn, Massachusetts, United States of America

RNA plays a wide variety of roles in every cell, from structural to informational to catalytic. That variety depends in large part on its ability to adopt a three-dimensional conformation suited to each application, often in tight association with protein or other polynucleotides. Those conformations are dynamic, and like proteins, RNAs are prone to misfolding as they convert from one to another. To cope with this problem, cells in all branches of life have evolved a set of RNA chaperone proteins, whose job it is to remodel the structure of their target RNAs, correcting mistakes and shepherding them into their correct shapes.

Among these chaperones are the so-called DEAD-box helicases, which bind to short double-helical sections of RNA, and, powered by ATP, unwind them. While much has been learned about these proteins, several important details of their mechanism of action remain unknown. Specifically, it has not been clear what role, if any, the helicase plays in undoing the tertiary contacts made between the target helix and other parts of the RNA molecule. In this issue of *PLOS Biology*, Cynthia Pan, Rick Russell, and colleagues demonstrate that one such helicase simply waits for those tertiary contacts to detach before capturing the helix, preventing rebinding and preparing it for unwinding.

The authors studied CYT-19, a DEAD-box protein from the bread mold *Neurospora crassa*, as it bound to and unwound a ribozyme from the protozoan *Tetrahymena thermophila*. The complex secondary structure of the ribozyme includes the so-called P1 helix, a target for CYT-19. As a consequence of binding its oligonucleotide substrate, P1 “docks” with other parts of the ribozyme, taking on a specific tertiary structure.

The authors interrogated the dynamics of the ribozyme's interactions with CYT-19 by using single-molecule Forster (or fluorescence) resonance energy transfer, or smFRET. In this technique, two different dyes are attached to each member of an interacting pair of molecules, in this case the ribozyme and its oligonucleotide substrate, which binds to the ribozyme to form the P1 helix. If the two dye molecules are close together, laser excitation of one of the dyes (the donor) causes light to be emitted from the other dye, the acceptor, due to resonance between the two dye molecules. The degree of separation of the two dyes can therefore be determined by monitoring the emitted light. The specific wavelength of the emission indicates whether it comes from the donor or acceptor dyes. With a single molecule approach, this information can be obtained in real time for each ribozyme molecule. In this experiment, when P1 assumed its full tertiary, docked conformation, most of the emitted light came from the acceptor, whereas when P1 was in the undocked position, most of the emitted light came from the donor. The unwinding of the P1 helix secondary structure led to the loss of all fluorescence, as the dye-labeled oligonucleotide was released into solution.

**Figure 1:**
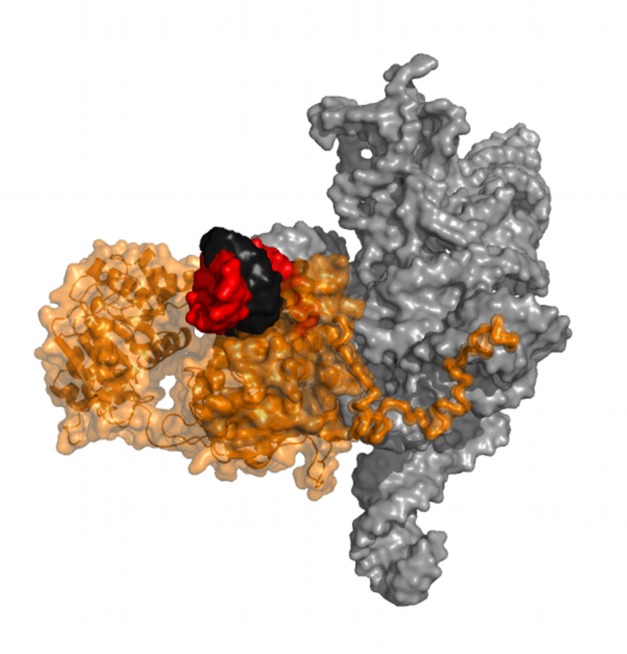
CYT-19 (orange) captures an RNA helix (red and black) after waiting for the helix to undock spontaneously from tertiary contacts formed with the group I intron core (gray).

The authors found that without CYT-19 present, the P1 helix underwent spontaneous conversion between the docked and undocked conformations, with the secondary structure of the helical segment remaining intact. When they added CYT-19 and ATP, helix unwinding commenced but was almost entirely from the undocked state. Adding more CYT-19 did not increase the rate of docked-to-undocked conversion, indicating that this transition was not facilitated by the helicase. Instead, the helicase in effect “waited” for the ribozyme to undock and then captured it in its undocked state (Figure 1).

Once the helix had undocked, binding of CYT-19 slowed its conversion back into the docked state, an effect that did not require ATP. This indicated that capture of the P1 helix did not involve the energy-driven closure of two of the enzyme's domains (ATP was required to unwind P1). Further experiments showed that CYT-19 remained bound to the ribozyme even after its helicase core disengaged from P1, most likely by employing a strongly basic and unstructured tail. The ability to remain tethered to the ribozyme facilitated the subsequent recapture of P1.

The same essential behavior was seen in the yeast DEAD-box protein, Ded1, which, like CYT-19, did not weaken tertiary contacts between P1 and other portions of the ribozyme but did slow their re-formation. Unlike CYT-19, Ded1 needed to have ATP in place for helix capture, although it did not require its hydrolysis; a nonhydrolyzable analog worked just as well.

The fact that two different DEAD-box proteins employ a similar capture strategy for their interaction with RNA helices suggests this may be a mechanism used elsewhere, not only for RNA chaperone functions but perhaps in synthesis of ribosomes and spliceosomes, both of which rely on DEAD-box proteins for conformational transitions during their assembly. Further investigations are likely to reveal the variations different helicases in different organisms have evolved to make use of this basic tool for RNA remodeling.

**Pan C, Potratz JP, Cannon B, Simpson ZB, Ziehr JL, et al. (2014) DEAD-Box Helicase Proteins Disrupt RNA Tertiary Structure Through Helix Capture.** doi:10.1371/journal.pbio.1001981

